# Effects of orally applied butyrate bolus on histone acetylation and cytochrome P450 enzyme activity in the liver of chicken – a randomized controlled trial

**DOI:** 10.1186/1743-7075-10-12

**Published:** 2013-01-22

**Authors:** Gábor Mátis, Zsuzsanna Neogrády, György Csikó, Anna Kulcsár, Ákos Kenéz, Korinna Huber

**Affiliations:** 1 Department of Physiology and Biochemistry, Faculty of Veterinary Science, Szent István University, István utca 2, H-1078, Budapest, Hungary; 2 Department of Pharmacology and Toxicology, Faculty of Veterinary Science, Szent István University, István utca 2, H-1078, Budapest, Hungary; 3 Department of Physiology, University of Veterinary Medicine, Bischofsholer Damm 15/102, D-30173, Hannover, Germany

**Keywords:** Short chain fatty acids, Butyrate, Histone deacetylase inhibitor, Histone hyperacetylation, Epigenetics, Cytochrome P450 enzymes

## Abstract

**Background:**

Butyrate is known as histone deacetylase inhibitor, inducing histone hyperacetylation *in vitro* and playing a predominant role in the epigenetic regulation of gene expression and cell function. We hypothesized that butyrate, endogenously produced by intestinal microbial fermentation or applied as a nutritional supplement, might cause similar *in vivo* modifications in the chromatin structure of the hepatocytes, influencing the expression of certain genes and therefore modifying the activity of hepatic microsomal drug-metabolizing cytochrome P450 (CYP) enzymes.

**Methods:**

An animal study was carried out in chicken as a model to investigate the molecular mechanisms of butyrate’s epigenetic actions in the liver. Broiler chicks in the early post-hatch period were treated once daily with orally administered bolus of butyrate following overnight starvation with two different doses (0.25 or 1.25 g/kg body weight per day) for five days. After slaughtering, cell nucleus and microsomal fractions were separated by differential centrifugation from the livers. Histones were isolated from cell nuclei and acetylation of hepatic core histones was screened by western blotting. The activity of CYP2H and CYP3A37, enzymes involved in biotransformation in chicken, was detected by aminopyrine N-demethylation and aniline-hydroxylation assays from the microsomal suspensions.

**Results:**

Orally added butyrate, applied in bolus, had a remarkable impact on nucleosome structure of hepatocytes: independently of the dose, butyrate caused hyperacetylation of histone H2A, but no changes were monitored in the acetylation state of H2B. Intensive hyperacetylation of H3 was induced by the higher administered dose, while the lower dose tended to increase acetylation ratio of H4. In spite of the observed modification in histone acetylation, no significant changes were observed in the hepatic microsomal CYP2H and CYP3A37 activity.

**Conclusion:**

Orally added butyrate in bolus could cause *in vivo* hyperacetylation of the hepatic core histones, providing modifications in the epigenetic regulation of cell function. However, these changes did not result in alteration of drug-metabolizing hepatic CYP2H and CYP3A37 enzymes, so there might be no relevant pharmacoepigenetic influences of oral application of butyrate under physiological conditions.

## Background

Short chain fatty acids (SCFA) are produced physiologically by the anaerobic microbial fermentation of dietary compounds in the rumen of polygastric animals and mostly fibers in the large intestine of monogastric mammals, birds and humans [1]. Butyrate is of special interest due to its numerous positive effects on the health of gut and extraintestinal tissues. Butyrate is the most important energy source of the colonocytes [2], regulating also the proliferation and differentiation of the gastrointestinal epithelium [3] and inducing apoptosis in genetically disordered cells [4,5]. As a consequence, butyrate has a protective effect against colorectal cancer, which was reported in some *in vitro*[6] and also *in vivo* animal studies [7,8]. Due to its selective antimicrobial action on most enteral pathogens [9,10], butyrate improves the balance of the intestinal microflora, which can influence the health of the host animal or the human host [11].

Fiber-rich diet or uptake of resistant starch increases microbial butyrate production, but butyrate is also orally applicable in several forms. In animal nutrition, due to its numerous beneficial properties improving health and also the growth performance of pigs [12] and chickens [13], butyrate is of special interest as a nutritional supplement, especially after the banning of the traditional antibiotical growth promoters in the European Union [14].

Furthermore, as an epigenetic factor, butyrate regulates the transcription via influencing core histone acetylation, which is one of the most relevant epigenetic regulations of the cell function together with DNA methylation [15]. The dynamic balance of acetylation of histone proteins at certain lysine residues is regulated by the opposing effects of histone acetyltransferases (HAT) and histone deacetylases (HDAC) [15]. Butyrate inhibits Class I and most of the Class II HDAC enzymes, causing histone hyperacetylation at lysine residues of the N-terminal tail, therefore modifying the expression of certain genes [16]. In addition to the several *in vitro* studies [17], increased total histone acetylation was reported in case of porcine caecal tissue after dietary supplementation with the butyrate precursor lactulose [18]. Butyrate-induced histone modifications may be highly involved in butyrate’s antitumor, antibacterial and metabolic effects [19].

Although butyrate is greatly metabolized by the intestinal epithelium, a certain amount is also absorbed into the portal blood [20] and taken up by the liver in rat and human *in vivo*[21,22]. Butyrate is an important energy source for the liver as a substrate of the oxidative pathways, but it is also a potent effector of the hepatic metabolism. It can decrease the mitochondrial oxidative phosphorylation yield and the ATP content of the liver due to its uncoupling-like effect [23,24] and can influence the mitochondrial ATP turnover linked to glycogen metabolism [25].

It is known that the expression of certain microsomal cytochrome P450 (CYP) monooxygenases, playing a predominant role in biotransformation, drug and steroid metabolism, can be affected by histone modifications [26]. For instance, the HDAC inhibitor trichostatin A was shown to influence the *in vitro* expression of the CYP3A subfamily [27]. Alimentary added inulin, which is fermented by the colonic bacteria to SCFA, alleviates the reduction in the expression and activity of hepatic CYP1A1/2 and CYP2E1 enzymes in rats kept on a high-fat diet [28], possibly due to the epigenetic effects of the absorbed SCFA. On the basis of these findings, the enteral microbiome-produced or the orally added butyrate may also alter the activity of CYP enzymes, have an impact on hepatic detoxification capacity and drug metabolism, defined as possible pharmacoepigenetic influences.

The present study aimed to evaluate the epigenetic effects of butyrate added orally to broiler chickens in a daily bolus. These animals have a large capacity of growing and intensive hepatic metabolism. Young chickens have quite low rates of butyrate production in the large intestine [29], so they can be proper candidates in order to study the effects of the orally applied butyrate. Unlike in our previous study with butyrate evenly mixed in the feedstuff of the chicken, butyrate administered in bolus after starvation provides a fast, but short-term release of greater amount of butyrate to the portal vein and an intensive stimulus for the liver. The lower dose of butyrate, 0.25 g/kg body weight (BW) was chosen regarding the usual applied concentration of butyrate as a nutritional supplement. With the higher administered concentration, 1.25 g/kg BW we aimed to provide high amount of butyrate for the hepatocytes to study also the dose-dependency of its action.

After butyrate treatments, at first we wanted to monitor the modifications in the acetylation state of hepatic core histones at the most frequent acetylation sites. Our second goal was to measure the activity of CYP2H and CYP3A37 enzymes to screen, whether butyrate in bolus can influence the detoxification capacity of the liver.

## Methods

### Chemicals

Chemicals were purchased from Sigma-Aldrich (Munich, Germany) except when otherwise specified.

### Animals

One-day-old broiler chicks of the Ross 308 strain (mixed gender) were obtained from a commercial hatchery (Bábolna Tetra Company, Uraiújfalu, Hungary). Animals were housed individually in metal pens in a room with controlled environment conditions of Ross technology [30]. Feed and water were provided *ad libitum*. The diet was formulated according to the requirements of the starter period and was free from any medication or chemical additives. Composition of the diet is shown in Table 1.

**Table 1:** Composition of the diet of chickens

**Item**	
**Ingredient, g/kg**	
Corn	593.7
Soybean meal	310.0
Corn gluten meal	50.0
Limestone	15.0
Monocalcium phosphate	18.5
NaCl	4.0
Vitamin-mineral mixture^1^	6.0
L-Lysine HCl	1.8
DL-Methionine	1.0
**Calculated nutrient composition**	
Crude protein, g/kg	212.2
Ether extract, g/kg	29.4
Crude fiber, g/kg	25.3
Ash, g/kg	65.9
AME, MJ/kg	11.9
Lysine, g/kg	11.9
Methionine, g/kg	4.9
Methionine + Cysteine, g/kg	8.6
Calcium, g/kg	11.6
Available phosphorus, g/kg	4.5

^1^Provided (per kilogram of diet): Se, 0.24 mg; Fe, 135 mg; Mn, 136 mg; Cu, 22 mg; Zn, 110 mg; I, 1.2 mg; retinyl acetate, 4.14 mg; cholecalciferol 0.075 mg; α-tocopherol, 26.85 mg, choline chloride, 360 mg; menadione, 3.0 mg; riboflavin, 7.0 mg; cobalamine, 0.03 mg; niacin, 40 mg; pantothenic acid, 12 mg; folic acid, 1.0 mg; pyridoxine, 5.0 mg.

All procedures were conducted in accordance with international and national laws and institutional guidelines and approved by the Local Animal Test Committee of the Faculty of Veterinary Science, Szent István University, Budapest, Hungary (number of permission: 22.1/4719/003/2008).

### Treatments

On days 20–24 experimental animals were fasted overnight for 12 h and thereafter treated once daily by a crop-tube with an intraingluvial bolus according to the following protocol: (i) ten chickens received 0.1 g/ml sodium butyrate solution (2.5 ml/kg BW, which equals 0.25 g sodium butyrate/kg BW daily, 1.25 g sodium butyrate/kg BW for the total treatment period, which is approx. 0.95 g/animal in average); (ii) ten broilers were treated with 0.5 g/ml sodium butyrate solution (2.5 ml/kg BW, which equals 1.25 g sodium butyrate/kg BW daily, 6.25 g sodium butyrate/kg BW for the total treatment period, which is approx. 4.75 g/animal in average); (iii) distilled water (2.5 ml/kg BW) was applied for ten chicks as a control group. The butyrate boli did not cause any macroscopic pathomorphological alterations in the mucosa of the gastrointestinal tract, which may have been caused by higher osmolarity of the applied solutions. In addition, (iv) six broilers were treated on days 20–24 by intracoelomal phenobarbital (PB) injection (Phenobarbital sodium, Ph. Eur. 7.1, dissolved in sterile, pyrogen-free and endotoxin-free physiological saline solution, applied dose: 80 mg/kg BW daily) to induce CYP activity as a positive control. Body weight was measured individually on each day of treatment, boli and PB injections were adjusted to the measured body weight per day. (Mean body weight of the animals was 0.683 ± 0.011 kg on day 20 and 0.774 ± 0.017 kg on day 24.) All animals were starved for additional 2 h after each treatment in order to enhance the absorption of butyrate. Daily body weight gain and feed intake matched the requirements of the Ross technology and no significant difference could be observed between the groups. However, the applied butyrate provided some extra energy for the treated animals (calculated mean metabolizable energy content of the boli was 2.8 kJ/animal daily at the lower and 13.9 kJ/animal daily at the higher dose), it appeared not to be relevant compared to the energy content of the diet (calculated mean metabolizable energy provided by the diet taken up was 1190 kJ/animal daily).

### Liver sampling and separation of subcellular organelles

Animals were slaughtered in carbon dioxide anaesthesia by decapitation on day 24. Last treatment was conducted 2 h prior to slaughtering. After opening the coelom, the liver was exsanguinated with chilled physiological saline solution through the portal vein and was ectomized, weighed and shock-frozen immediately in liquid nitrogen.

After thawing, cell nucleus fraction was isolated from the liver of the bolus-treated and control chickens (treatments i, ii, iii) in order to examine the acetylation state of the core histones, while microsomal fractions were prepared from all animals (treatments i, ii, iii, iv) to study the hepatic CYP activity.

Subcellular organelles were isolated by differential centrifugation according to the protocol of Van der Hoeven [31]. Microsomal total protein concentration was determined with a Bicinchoninic Acid Protein Assay Kit (Fisher Scientific, Rockford, IL, USA) on a microplate in triplicates, using bovine serum albumin (BSA) as a standard.

All cell nucleus and microsomal fractions were shock-frozen in liquid nitrogen and were stored at −80°C until further examinations.

### Histone isolation

Purified histone extracts were isolated by a Histone Purification Mini Kit (Active Motif, Carlsbad, CA, USA) from cell nucleus fractions according to the manufacturer’s protocol. During the whole purification procedure kit reagents prevented further deacetylase activity to ensure acetylation status as *in vivo*.

Equal volume of ice-cold Extraction Buffer was added to the nucleus suspension. After homogenization, samples were incubated overnight at 4°C on a rotating platform. Tubes were centrifuged at maximum speed (30,000 g) for 5 min in a microfuge, and the supernatant, considered as the crude histone extract, was neutralized with one-fourth volume of 5x Neutralization Buffer (pH 8.0). Neutralized extract was loaded onto previously equilibrated histone isolation spin columns. After 3 washing steps with Wash Buffer, histones were eluted and precipitated overnight from the flow-through by 4% perchloric acid. Precipitate was sedimented by centrifugation at 30,000 g for 60 min, the pellet was washed at first with 4% perchloric acid, later with acetone, containing 0.2% HCl and finally with pure acetone. Histones were resuspended in sterile distilled water and the yield of total core histone proteins was quantified by measuring the absorbance at 230 nm.

### Western blot analysis

Electrophoresis and western blotting were performed according to the instructions of the applied Acetyl Histone Antibody Sampler Kit (Cell Signaling, MA, USA). Histone preparations were diluted by SDS- and mercaptoethanol-containing loading buffer (supplemented with 50 mM dithiothreitol), sonicated for 15 sec in order to reduce viscosity and proteins were heat denatured at 95°C for 5 min. Histones were separated by SDS-PAGE on polyacrylamide (4-20%) precast gradient gels (Biorad Laboratories, CA, USA), the amount of loaded protein was 3 μg per lane for the detection of histones H2A and H3, while 6 μg per lane for histones H2B and H4. After tank blotting of proteins onto nitrocellulose membranes (0.2 μm pore size, Biorad Laboratories, CA, USA), histones were identified by immunodetection using antibodies of the Acetyl Histone Antibody Sampler Kit: after blocking with 5% fat-free milk-containing PBST for 2 h, the immunoblots were incubated overnight with primary antibodies against histone H2A (1:1000), H2B (1:500), H3 (1:1000), H4 (1:500) and their acetylated forms. Each acetyl histone antibody was specific for the target histone modified at the lysine residue of the most frequent acetylation site (H2A and H2B: Lys 5, H3: Lys 9, H4: Lys 8). Detection of the primary antibody was performed using an anti-rabbit secondary antibody (1:2000) coupled with horseradish peroxidase. Primary antibodies were diluted in PBST, containing 5% BSA, while secondary antibodies in PBST, containing 5% fat-free milk. Bands were detected by the Chemidoc XRS enhanced chemiluminescence system (Biorad Laboratories, CA, USA). Membranes were finally stained by Indian Ink to detect all the separated proteins. Band intensities were quantified by the Quantity One 1-D Analysis software (Biorad Laboratories, CA, USA), trace quantities were standardized to the Indian Ink stained bands to ensure equal loading. Acetylation ratios were determined considering relative protein expression levels of each histone and its acetylated form.

All western blot examinations were carried out in duplicates. Regarding histone H3, due to their different molecular mass, trace quantities of bands representing H3.1 and H3.2 isoforms could be measured separately, but the acetylation state was calculated from the total amount of H3 and acetyl-H3.

### Enzyme assays on hepatic microsomal CYP activity

#### Aminopyrine N-demethylation assay

Microsomal CYP2H/CYP3A37 activity was screened by the aminopyrine N-demethylation assay, in which formaldehyde production could be measured by the spectrophotometric method of Nash [32]. The enzyme assay was performed according to the modified protocol of García-Agúndez et al. [33]. The reaction mixture contained an NADPH+H^+^-regenerating cofactor mixture, prepared from 0.5 mM NADPH+H^+^ (Reanal Private Ltd., Budapest, Hungary), 50 mM glucose 6-phosphate, 4 IU/l glucose 6-phosphate dehydrogenase, 5 mM MgCl_2_ and 50 mM semicarbazide.

After thawing on ice, 100 μl microsomal suspension was incubated with 200 μl cofactor mixture and 900 μl 0.05 M phosphate buffer (pH 7.4) in the presence of different concentrations (0, 1.25, 2.5, 5, 10 mM) of dimethylamino-antipyrine (aminopyrine) for 10 min at 37°C. The reaction was stopped by adding 200 μl 20% trichloroacetic acid. After centrifugation at 4,500 g for 10 min, 400 μl Nash reagent (0.16 ml acetyl acetone and 0.24 ml concentrated acetic acid in 20 ml of 4 M ammonium acetate solution) was added to 800 μl of the supernatant. The mixture was incubated at 60°C for 30 min, cooled down on ice and the absorbance was measured spectrophotometrically at 415 nm against reagent blank. Results were corrected by subtracting the absorbance of an inhibited blank per each substrate concentration (inhibited previously by adding 20% trichloroacetic acid). Formaldehyde standard curves were determined under the same conditions as used for microsomal activity measurements; each sample was examined in triplicates. Finally, mean specific enzyme activity (reaction velocity), maximal reaction velocity (V_max_) and the Michaelis-Menten’s constant (K_M_) were calculated and compared between groups. All results were standardized according to the total protein concentration of microsomal samples.

#### Aniline-hydroxylation assay

CYP2H activity was measured by aniline-hydroxylation assay. The enzyme assay was carried out according to the modified protocol of Murray and Ryan [34]. The reaction mixture contained an NADPH+H^+^-regenerating cofactor mixture with the same composition as for the aminopyrine N-demethylation assay. After thawing on ice, 100 μl microsomal suspension was incubated with 200 μl cofactor mixture and 900 μl 0.05 M phosphate buffer (pH 7.4) and different concentrations (0, 1.25, 2.5, 5, 10 mM) of aniline hydrochloride for 15 min at 37. The reaction was terminated by adding 200 μl 20% trichloroacetic acid. Following centrifugation at 4,500 g for 10 min, 400 μl 10% Na_2_CO_3_ solution and 400 μl alkaline phenol solution (0.8% phenol solution in 0.2 M NaOH) were added to 400 μl of the supernatant. The mixture was incubated at 37°C for 30 min, cooled down on ice and the absorbance was measured by spectrophotometer at 605 nm against reagent blank. An inhibited blank was approved for each substrate concentration similarly to the aminopyrine N-demethylation assay. To determine the amount of the produced 4-aminophenol, standard curves were prepared; each sample was examined in triplicates. Mean specific enzyme activity, V_max_ and K_M_ values were also determined and compared between groups. All results were standardized according to the total protein concentration of microsomal samples.

### Statistics

All values are expressed as means ± SEM. Statistical analysis of data was performed with R 2.14.0 software (downloaded from http://cran.r-project.org/bin/windows/base/old/2.14.0/ on 14 December 2011), one-tailed non-parametric Mann-Whitney’s test and one-way ANOVA were approved for comparison of results of the treated groups with those of controls. Level of significance was set at P<0.05.

## Results and discussion

### Acetylation of hepatic core histones

**Figure 1:**
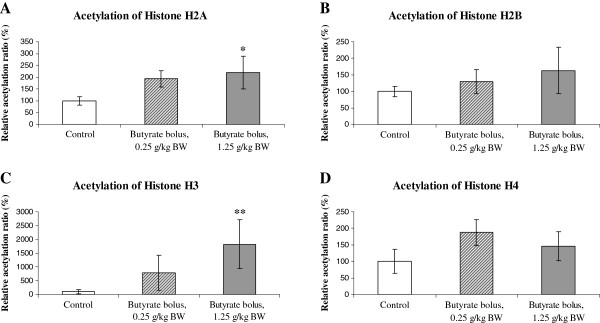
**A-D. Relative acetylation of hepatic core histones in chicken after oral application of butyrate bolus.** Butyrate was applied at a lower (0.25 g/kg BW) and a higher (1.25 g/kg BW) dose, relative acetylation ratios were compared to those of controls (considered as 100%). Acetylation ratios were determined considering relative protein expression levels of each histone and its acetylated form. Data are presented as mean ± SEM (n=6/group). ^*^Significant difference, P<0.05; ^**^Significant difference, P<0.01.

**Figure 2:**
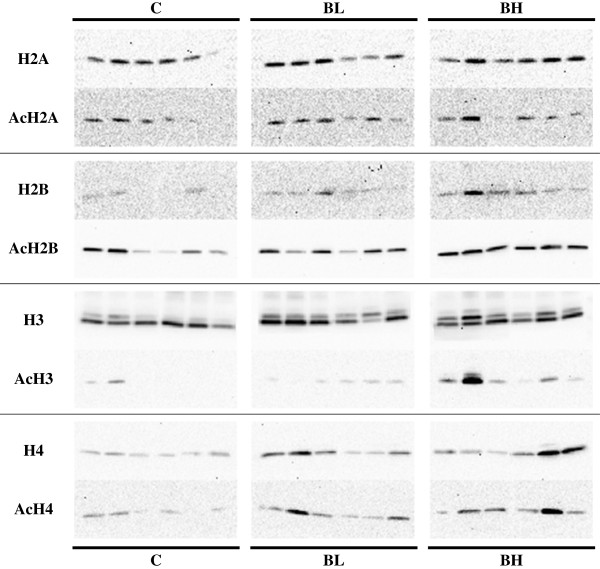
**Representative bands as obtained by western blotting from isolated hepatocyte histones of chickens.** Columns show the bands of control animals (C) and chicks after oral application of butyrate bolus at the dose of 0.25 g/kg body weight (BL) and the dose of 1.25 g/kg body weight (BH). The upper rows show the relative protein expression levels of total H2A, H2B, H3 and H4, respectively, while the bands specific for acetylated histones of the same animals can be seen below (AcH1-4). At histone H3, the upper band can be identified as the H3.1 isoform and the lower as H3.2. Western blots were done in duplicates for all histones.

Screening of the important acetylation sites of core histones showed that butyrate treatment in bolus at the lower dose (0.25 g/kg BW) tended to increase acetylation of histone H2A at lysine 5 (P=0.063), and the higher applied dose (1.25 g/kg BW) caused significant, approximately twofold increase in acetylation (P=0.048) compared to the control group at the same acetylation site of H2A (Figure 1A and Figure 2).

Confirming this finding, butyrate induced hyperacetylation of H2A in colonic epithelial cell culture *in vitro*[35]. Acetylation of H2A is of special importance since its acetylation state is highly involved in conformational changes of the nucleosome and decreased histone-DNA interactions, working synergistically with acetylation of the N-terminal histone tails [36,37].

In contrast, butyrate bolus did not influence the acetylation of histone H2B at lysine 5 with the lower (P=0.274) nor the higher dose of butyrate (P=0.714) (Figure 1B and Figure 2). There are still some other lysine residues in H2B, which may be potential targets of HDAC inhibitors [38], and possible effects of butyrate on these other acetylation sites cannot be excluded.

There was no significant difference in the acetylation ratio of total histone H3 at lysine 9 after the application of butyrate in the lower dose (P=0.146). However, higher dose of butyrate caused relevant, approximately 18-fold increased H3 acetylation ratio (P=0.009) (Figure 1C and Figure 2). Hyperacetylation of H3 after butyrate exposure was reported by several *in vitro* studies in a variety of cultured mammalian cells, but not yet described *in vivo*. It was found already in 1973 that butyrate in millimolar concentrations caused hyperacetylation of H3 and H4 in all examined vertebrate cell lines, while H2A and H2B were also affected in certain rat-derived cell cultures [17]. Butyrate-induced dynamic histone acetylation was compared between mammalian and avian cells *in vitro*[16], where huge amount of highly acetylated H3 isoforms was found after butyrate treatment in human breast cancer cells, in contrast of terminally differentiated avian immature erythrocytes, 2% of which participated in the acetylation process. Among the many acetylation sites, in agreement with our results, it was recently described that butyrate induced H3 hyperacetylation first of all at lysine 9, an acetylation site that plays a critical role in the epigenetic regulation of cell function [39]. Since this acetylation site is linked to histone phosphorylation and methylation processes, these site-specific modifications together can cause distinct chromatin alterations and cell cycle modifications [40].

The H3 isoforms H3.1 and H3.2 could be also separated on the immunoblots and it was found that butyrate increased the relative protein expression level of the H3.1 isoform, which was poorly expressed in control animals, but was detected in high amount in both butyrate-treated groups. The difference in the relative protein expression level of H3.1 between the control and butyrate-treated groups was considered to be significant after the application of the lower dose (P=0.021) and a near-significant trend following treatment with the higher dose (P=0.090) of butyrate. It is known that three H3 variants (H3.1, H3.2, H3.3) do exist in mammals, specifically, H3.1 is involved in both chromatin activation and repression, while H3.2 plays an important role in gene repression and H3.3 is especially enriched in active marks [41]. Unlike in the case of mammals, only H3.1 and H3.2 could be separated from chicken cells [42]. Due to the pleiotropic effect of H3.1 on transcription, increased protein expression level of H3.1 after butyrate treatment, detected in our present study, may be also of special importance.

Regarding the acetylation of histone H4 at lysine 8, butyrate tended to induce hyperacetylation at the lower administered dose (P=0.063) (Figure 1D and Figure 2). Similarly to H3, H4 is also a highly involved target of butyrate-induced hyperacetylation in cell cultures [17]. It is known that acetylation and deacetylation of H4 is a well-coordinated process, and butyrate-induced tetra- and tri-acetylated forms of H4 are always acetylated at lysine 8 [38]. Therefore, the lysine residue examined in this study is considered as one of the most important acetylation sites of H4. It was recently also stated that H3 at lysine 9 and H4 at lysine 8 are critical targets of butyrate-induced histone hyperacetylation, which process is associated with the G-protein-coupled receptor-41, also activated by butyrate [43]. Interestingly, the acetylation ratio of H4 after force-feeding with the higher dose of butyrate was not increased significantly compared to the control group (P=0.210) (Figure 1D and Figure 2). Butyrate can also alter the activity of HAT enzymes, and this contradictory finding may be in association with the pleiotropic effects of butyrate on HAT and HDAC [44], depending also on the dose of butyrate.

Very little data can be found in literature regarding the *in vivo* effects of butyrate on the chromatin structure. In a recent study, significant increase in total histone acetylation was reported in the caecal tissue of pigs after receiving orally administered lactulose, which was intensively fermented to butyrate in the large bowel [18].

It is also interesting to compare the present results after butyrate application in bolus with our recent experiment [45], where butyrate was applied as a nutritional supplement for broiler chickens for three weeks at the concentration of 1.5 g/kg diet. Dosage of butyrate as a supplement was approximately equivalent with the lower dose bolus (0.25 g/kg BW) in the present study. The most important difference between the treatments is that the administration of butyrate in bolus after overnight starvation provides a short-term supply of greater amount of butyrate for the hepatocytes. In the earlier experiment animals could take up butyrate-supplemented diet the whole day, but this uptake might be followed by a prolonged absorption and a long-acting butyrate exposure of the liver.

In both experiments, hyperacetylation of H2A at lysine 5 was found and no dose-dependency could be detected after bolus treatment. In spite of these results, it can be stated that butyrate did not affect the acetylation state of histone H2B at lysine 5 after butyrate administration in bolus, nor in the nutritional supplement study. Similarly to our earlier experiment, where butyrate was applied as a nutritional supplement, the lower dose of butyrate bolus did not cause any changes in the acetylation of histone H3 at lysine, but the higher dose induced a highly relevant hyperacetylation of H3 at lysine 9. Due to the key-role of H3 modifications in gene expression [39,40], this action seems to be a very important change in the epigenetic regulation of transcription. Lower dose of butyrate tended to increase acetylation of H4 at lysine 8, differing from the nutritional supplement experiment, underlining the critical role of the application.

We can summarize that orally applied butyrate in bolus influenced hepatic histone acetylation *in vivo*, core histones H2A, H3 and H4 were involved in this partly dose-dependent action. Since butyrate modified the chromatin structure, it can be considered as an important epigenetic effector on gene expression of hepatocytes. Therefore, hepatic CYP activity was measured as key surrogate to assess changes in gene expression and subsequently, function.

### Activity of hepatic microsomal CYP enzymes

We hypothesized that butyrate-induced histone modifications might cause changes in hepatic CYP activity; because it was reported that histone acetylation had a huge impact on gene expression of several members of the CYP2 family [26]. It is also known that alterations in histone H3 acetylation are involved in the expression of CYP3A subfamily in the adult mouse [46]. However, in our experiment, butyrate-induced epigenetic changes of chromatin structure did not result in changes of enzyme activity.

**Figure 3:**
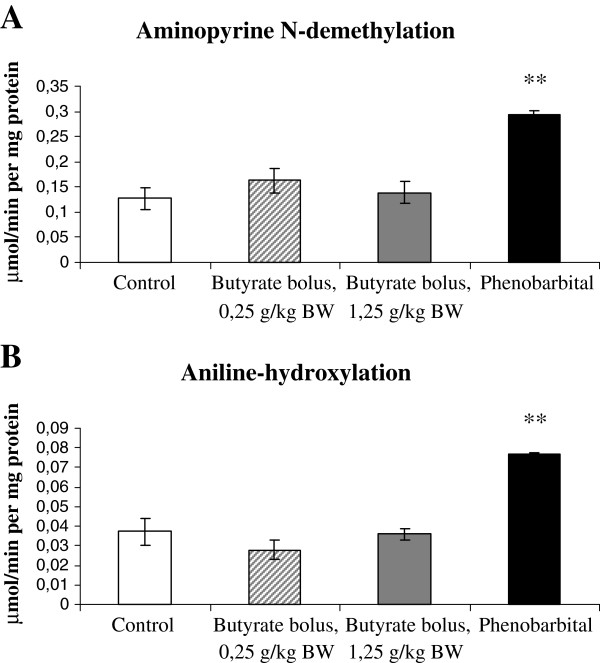
**Mean specific activity of hepatic microsomal CYP enzymes of chickens. A.** Average amount of formaldehyde produced in aminopyrine N-demethylation assay (μmol per minute per mg microsomal protein), indicating the mean specific activity of hepatic microsomal CYP2H/CYP3A37 isoenzymes. **B.** Average amount of 4-aminophenol produced in aniline-hydroxylation assay (μmol per minute per mg microsomal protein), indicating the mean specific activity of hepatic microsomal CYP2H isoenzyme. Results of enzyme assays, carried out with the hepatic microsomal fraction of chickens after oral application of butyrate bolus at a lower (0.25 g/kg BW, n=10) and a higher (1.25 g/kg BW, n=9) dose or treated with intracoelomal phenobarbital (PB) injection (80 mg/kg BW, n=6), are compared to those of controls (n=9). Data are presented as mean ± SEM. ^**^Significant difference, P<0.01.

Screening the aminopyrine N-demethylation activity of hepatic microsomal fractions, catalyzed by CYP2H and CYP3A37 enzymes, no significant difference was found between the mean enzyme activity (reaction velocity) of the butyrate-treated animals and those of controls (lower dose: P=0.196, higher dose: P=0.523) (Figure 3A). Similarly, administration of butyrate bolus caused no significant differences in the V_max_ values, independently of the applied dose (lower dose: P=0.368, higher dose: P=0.911). The lower concentration of butyrate did not affect the K_M_ value (P=0.713), but the higher dose tended to decrease it (P=0.095), however, due to high standard error of mean it can be considered only as a near-significant trend (Table 2).

**Table 2:** **Effects of oral application of butyrate on kinetic properties of cytochrome P450 (CYP) enzymes in chicken**^**1**^

**Enzyme action**	**Responsible CYP**	**Kinetic parameter**	**Control**	**Butyrate 0.25 g/kg BW**^**2**^	**Butyrate 1.25 g/kg BW**^**2**^	**PB**^**3**^
Aminopyrine N-demethylation	CYP2H/3A37	V_max_ (μmol/mg/min)	0.22 ± 0.06	0.21 ± 0.04	0.17 ± 0.03	0.38 ± 0.01^**^
		K_M_ (mM)	1.07 ± 0.24	1.20 ± 0.33	0.57 ± 0.15	1.02 ± 0.14
Aniline-hydroxylation	CYP2H	V_max_ (μmol/mg/min)	0.11 ± 0.02	0.15 ± 0.04	0.13 ± 0.03	0.19 ± 0.01
		K_M_ (mM)	5.27 ± 0.48	12.25 ± 4.36	7.86 ± 2.55	3.27 ± 0.45

^1^Values are shown as means ± SEM (control: n=9; butyrate, lower dose: n=10; butyrate, higher dose: n=9; PB: n=6).

^2^Butyrate = chickens treated with oral application of butyrate bolus in a lower (0.25 g/kg BW) and a higher (1.25 g/kg BW) dose.

^3^PB = chickens received intracoelomal phenobarbital injection (80 mg/kg BW) on days 20–24.

^**^Significant difference, P<0.01.

As an enzyme inductor, PB treatment caused notable enzyme induction with significantly increased mean reaction velocity (P=0.003) (Figure 3A) and V_max_ values (P=0.009), but did not influence the K_M_ (P=0.878) of the reaction (Table 2).

In agreement with these results, butyrate treatment in bolus did not alter the aniline-hydroxylation activity of the liver, specific for the microsomal CYP2H subfamily. No significant difference was found in the mean enzyme activity (lower dose: P=0.211, higher dose: P=0.848) (Figure 3B), V_max_ (lower dose: P=0.700, higher dose: P=0.640) and K_M_ (lower dose: P=0.354, higher dose: P=0.542) values (Table 2) between control and butyrate-stimulated chickens, independently of the applied dose.

PB treatment enhanced significantly the CYP2H activity: increased mean reaction velocity (P=0.002) was measured (Figure 3B), but V_max_ (P=0.267) and K_M_ values were not affected (P=0.760) (Table 2).

These data are in agreement with our previous results [45], where we found that butyrate as a feed supplement did not alter the activity of hepatic CYP2H and CYP3A37 enzymes in chicken. Now, on the basis of our previous and recent results, it can be stated, that independently of the form of application and the applied dose, alimentary butyrate did not modify the activity of the examined CYP enzymes under physiological conditions.

However, butyrate’s potential effects on other CYP subfamilies cannot be excluded. It is also not clear, whether under special dietary conditions or simultaneously applied with other agents, such as xenobiotics, butyrate may modify the liver enzymes of biotransformation. For example, dietary supplementation of inulin, a precursor of colonic butyrate production, in rats, suffering from high-fat-diet-induced hyperlipidaemia and hepatic steatosis, counteracted the decrease in the expression and activity of hepatic CYP1A1/2 and CYP2E1 enzymes [28].

## Conclusion

The present study indicated that (1) orally applied butyrate in bolus had an impact on chromatin structure of hepatocytes in chicken in the early post-hatch period: independently of the dose, butyrate caused hyperacetylation of histone H2A, but no changes were observed in the acetylation state of H2B. The higher administered dose (1.25 g/kg BW) induced an intensive hyperacetylation of H3, while the application of the lower dose (0.25 g/kg BW) tended to increase acetylation ratio of H4. In addition, both treatments with butyrate bolus increased the expression of the H3.1 isoform. (2) These changes in the epigenetic regulation of cell function did not result in alteration of drug-metabolizing hepatic CYP2H and CYP3A37 enzyme activity, so there might be no relevant pharmacoepigenetic influences of orally applicated butyrate under physiological conditions.

Results of this and former studies indicate that growing chicken are a suitable model to evaluate the epigenetic effects of orally applied butyrate both by diet and by intra-crop application. Due to low endogenous butyrate production the histone acetylation is most likely derived from exogenous butyrate directly. Although this is a strictly descriptive study, this chicken model provides a high potential to identify butyrate-induced epigenetic mechanisms and their consequences in metabolic regulation in future.

## Abbreviations

AME: Apparent metabolizable energy; ATP: Adenosine triphosphate; BSA: Bovine serum albumin; BW: Body weight; CYP: Cytochrome P450; EDTA: Ethylene diamino tetraacetic acid; HAT: Histone acetyltranferase; HDAC: Histone deacetylase; K_M_: Michaelis-Menten’s constant; NADPH+H^+^: Nicotinamide adenine dinucleotide phosphate (reduced form); PAGE: Polyacrilamide gelelectrophoresis; PB: Phenobarbital; PBST: Phosphate buffered saline supplemented with Tween; SCFA: Short chain fatty acids; SDS: Sodium dodecylsulphate; V_max_: Maximal reaction velocity.

## Competing interests

The authors declare that they have no competing interests.

## Authors’ contributions

GM designed and carried out animal treatments and sampling, prepared the cell nucleus and microsomal fractions, conducted western blot examinations and enzyme assays and drafted the manuscript. ZsN conceived and designed the study, carried out sampling and revised the manuscript, GyCs designed the study, carried out sampling, analyzed data and revised the manuscript, AK carried out animal treatments and sampling, conducted enzyme assays and analyzed data. ÁK carried out sampling and prepared the cell nucleus and microsomal fractions, KH conducted the western blot examinations, analyzed data and revised the manuscript. All authors read and approved the final version of the manuscript.
